# *eyeScrollR*: A software method for reproducible mapping of eye-tracking data from scrollable web pages

**DOI:** 10.3758/s13428-024-02343-1

**Published:** 2024-02-12

**Authors:** Nathanael Larigaldie, Anna Dreneva, Jacob L. Orquin

**Affiliations:** 1 Aarhus University, Aarhus C, Denmark; 2 Reykjavik University, Reykjavik, Iceland

**Keywords:** Eye tracking, Gaze mapping, Scrollable web pages, Software, Open source

## Abstract

The Internet has become an important part of our lives and an increasing number of researchers use eye-tracking technology to examine attention and behavior in online environments. Researchers, however, face a significant challenge in mapping eye-tracking data from scrollable web pages. We describe the R package *eyeScrollR* for mapping eye-tracking data from scrollable content such as web pages. The package re-maps eye-tracking gaze coordinates to full-page coordinates with a deterministic algorithm based on mouse scroll data. The package includes options for handling common situations, such as sticky menus or ads that remain visible when the user scrolls. We test the package’s validity in different hardware and software settings and on different web pages and show that it is highly accurate when tested against manual coding. Compared to current methods, *eyeScrollR* provides a more reproducible and reliable approach for mapping eye-tracking data from scrollable web pages. With its open code and free availability, we recommend *eyeScrollR* as an essential tool for eye-tracking researchers, particularly those who adhere to open-science principles. The *eyeScrollR* package offers a valuable contribution to the field of eye-tracking research, facilitating accurate and standardized analysis of eye-tracking data in web scrolling contexts.

In 2021, the average rate of Internet access exceeded 90% in OECD countries (OECD, 2021), and especially young people spend an increasing amount of time online (Anderson et al., 2018). The Internet has thus become an important part of our lives and an increasing number of researchers use eye-tracking technology to examine attention and behavior in online environments, for instance, in user experience (Lewandowski & Kammerer, 2021), online retailing (Tupikovskaja-Omovie & Tyler, 2021; Ladeira et al., 2019), advertising (Kaspar et al., 2019), and online education (Alemdag & Cagiltay, 2018).

Many of these studies, however, do not actually use web pages in their research but rely on static images of web pages or images of specific web page elements (Huddleston et al., 2015; Kanaan & Moacdieh, 2021; Luo, 2021; Schröter et al., 2021). While there may be several advantages to using images of web pages rather than actual web pages, the approach reduces ecological validity since participants cannot interact with or scroll up and down an image of a web page. The misalignment between research design and reality is most likely owing to the complexity of recording and processing eye-tracking data in online environments. Current methods face a significant challenge when it comes to mapping eye-tracking data to scrollable web pages. Typically, eye tracking on web pages will result in a video file of the participant browsing and scrolling through web pages with superimposed eye movements. However, at the analysis stage, the gaze position in the video recording is usually mapped to areas of interest (AOIs) on the web page. There are two main methods for processing eye movements in online settings.

The first method entails manually mapping fixations in the video recording to AOIs on a reference image of the web page (Holmqvist et al., 2011). The method is similar to how mobile eye-tracking data is usually coded with the key exception that in mobile eye tracking the reference image is a photograph of the environment that participants are navigating (e.g., Gidlöf et al., 2013).

The manual mapping method entails recording the screen while participants perform the study and afterwards manually mapping each gaze and/or fixation coordinate on each video frame to its corresponding location on a reference image of the web page. When there are a large number of participants, web pages, or AOIs in a study, the manual method becomes extremely time-consuming. Another disadvantage of manual coding is that it may be difficult to replicate due to subjective judgments about the precise location of fixations in relation to the reference image.

The second method entails using commercial eye-tracking software such as Gazepoint, Tobii, WebLink, or iMotions, for automatic or assisted gaze-mapping to coordinates on a reference image. This method, however, can also have a number of significant drawbacks. First, these software programs can be prohibitively expensive, limiting researchers’ access to the method. Second, since this software sometimes relies on heuristic computer vision algorithms, it does not always map gaze coordinates with satisfying accuracy, and in some situations, it can be unpredictable and unreliable, making it difficult to anticipate when or why the software will or will not work. Third, commercial software typically uses its own closed source code, which limits transparency and reproducibility since other researchers need access to exactly the same software version to reproduce the study. Finally, the majority of this software has user-friendly but imprecise interfaces (e.g., drag-and-drop boxes to define AOIs). As a result, even the most diligent researcher using the same software and the same version may be unable to exactly reproduce the study.

In addition, similar to manual coding, most commercial gaze-mapping software is based on a video recording of the screen. However, monitors typically refresh at least twice as often per second as video recordings which significantly reduces the maximum possible accuracy of the mapping process. Furthermore, online screen capturing is computationally expensive, and fluctuations in available computational power may cause occasional frames to lag or be skipped entirely, generally without the user’s knowledge. As a result, video recordings are untrustworthy representations of what was presented on the monitor during a study.

We aimed to develop a third method for processing eye-tracking data from real-world online environments. The purpose of this article is to introduce the novel method and the *eyeScrollR* software, an open-source R package (R Core Team, 2022) that provides researchers with a free, reproducible, and reliable method for mapping eye-tracking data to web pages. The *eyeScrollR* package re-maps eye-tracking gaze coordinates on the screen (e.g., screen dimensions 1920*1080 pixels) to coordinates on a full-page reference image (e.g., web page dimensions 1920*5000 pixels), by correcting the gaze *y*-coordinate up or down as a function of participant-generated actions like mouse scrolling. Similar to manual coding and commercial software, the gaze-mapped *eyeScrollR* data can be analyzed with AOIs. Unlike the two other methods, gaze-mapping with the *eyeScrollR* package makes it completely transparent, where changes have been made to the data allowing researchers to go beyond AOI-based analyses to, for instance, examine scan paths, saccadic amplitudes, micro-saccades, etc. The *eyeScrollR* package relies on a deterministic rather than heuristic algorithm, and it allows researchers to control any stage of data processing, forecast when and where the approach may or may not be employed with dependability, and improve it at will.

**Fig. 1:**
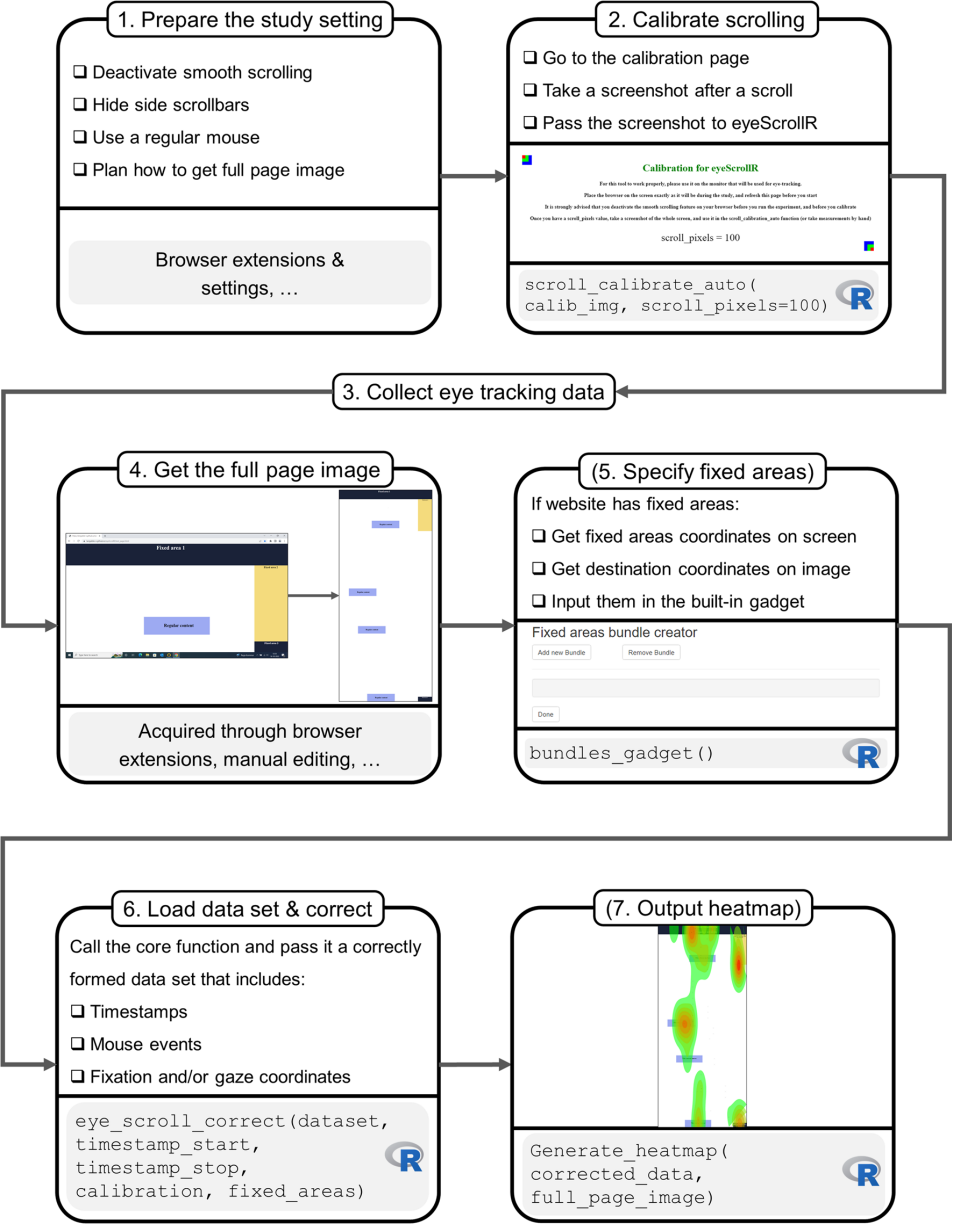
The typical seven steps of workflow when using the *eyeScrollR* package. Steps 5 and 7 are optional

## Package description and usage

*eyeScrollR* is an R package designed for researchers who wish to apply eye-tracking methods to real-world online environments. The package implements a gaze-mapping method that transforms eye-tracking gaze coordinates with screen dimensions to web page dimensions. The package can be used with any type of scrollable stimuli but is primarily intended for eye tracking on web pages. Its core function (*eye_scroll_correct*) loops through a chronologically ordered dataset containing as a minimum timestamps, participant-generated scroll events (such as key presses or mouse scroll events), and either gaze coordinates, fixation coordinates, or both. In other words, *eyeScrollR* reads through the dataset line by line, looking for moments when the user turned the scroll wheel and then applied an offset to all subsequent *y*-coordinates when applicable. For example, if *eyeScrollR* detects a down scroll worth 100 pixels, all subsequent gaze and fixation *y*-coordinates are increased by 100 pixels; however, this does not occur if the web page content has already been scrolled down to the very bottom. The method effectively allows the researcher to convert gaze or fixation data measured in terms of screen dimensions to web page dimensions. For interpreting the gaze-mapped data, it is useful to collect reference images in the form of full-page images of the web page which can be gathered with either dedicated software or browser plug-ins. The gaze-mapped data can then be superimposed on the full-page image as a heatmap.

Web pages often contain fixed areas that remain visible when the user scrolls and these areas may even change dynamically as a result of the scroll (e.g., a top navigation menu or a search bar that shrinks or disappears after the user has scrolled down a certain number of pixels). Such areas often remain in the same position on the screen and are therefore affected differently by scrolling. Consequently, gaze coordinates on these areas are not mapped in the same way as gaze coordinates on the regular web page content. The *eyeScrollR* package contains a function for handling fixed areas but it requires input from the researcher concerning the exact location of the fixed areas. The entire procedure can be summarized in a seven-step workflow, as shown in Fig. 1. The following sections describe these steps in detail and provide a complete reproducible example including commented code to be used in R.

### Step 1. Prepare the study setting

Reliable and accurate gaze-mapping with *eyeScrollR* depends on the usefulness of the recorded scroll data. It is therefore important that participants use a mouse with a scroll wheel that has tactile notches, where each notch of scroll corresponds to a fixed amount of scrolling on the screen. This means that participants cannot use navigation with analog scroll, such as an Apple mouse or laptop track pads. To ensure useful and unambiguous scroll data, it is also necessary to change certain browser settings before the data collection begins. Omitting correct browser settings may result in a few pixels of inaccuracy in the fixation coordinates, and the mapping may be temporarily ahead by up to 150 ms during scrolling blur periods. Omitting correct browser settings may occasionally corrupt the entire file, if, for instance, a participant has a very fast and erratic scrolling pattern. As a result, it is recommended to disable smooth scrolling in the browser and remove the side scrollbar. Deactivating smooth scrolling in Google’s Chrome browser can be done by typing ’chrome://flags/’ in the address bar and disabling the ’Smooth Scrolling’ option. A similar setting can be achieved in all Chromium-based browsers (e.g., Edge, Opera, etc.), and the Firefox settings menu includes an option to deactivate it. It is also recommended to hide or deactivate the side scrollbar. In Chrome/Chromium-based browsers, activating the ’Overlay Scrollbars’ option in the same menu is sufficient, while free browser extensions can be downloaded for Firefox browsers.

Finally, it is important to plan in advance if, how, and when full-page images of web pages will be gathered. Web pages with highly dynamic content may make it difficult to collect web page images before or after the data collection (e.g., social media web pages). Less dynamic content may allow researchers to collect the image right after eye-tracking data collection (e.g., online shopping, where the likelihood of products appearing or disappearing in a matter of seconds is low), while images of completely static web pages can be collected even before the eye-tracking data collection has begun.

### Step 2. Calibrate scrolling

*eyeScrollR* must be calibrated to the screen and the browser used during the eye-tracking study. Essentially, the calibration consists of gathering three pieces of information: a) the screen resolution, b) the exact coordinates of the top leftmost and bottom rightmost pixels of the viewing area (the visible part of the web page), and c) the number of pixels being scrolled whenever the user scrolls up or down one notch on the scroll wheel. This information is passed to the *eye_scroll_correct* function in step 6 and it informs the software about the number of pixels to correct for each mouse scroll event, and about where the scrollable content is placed on the screen. Calibration can be done automatically or manually. Automatic calibration requires making a screenshot of the calibration page (https://larigaldie-n.github.io/eyeScrollR/calibration.html) using the browser for which settings were prepared in Step 1 and then loading the screenshot image into R. The calibration function *scroll_calibration_auto* uses the image to compute all relevant calibration information. The calibration web page includes colored squares in the top left and bottom right corners of the viewing area, which the automatic calibration function detects in order to get the coordinates of the top leftmost and the bottom rightmost pixels of the viewing area. Screen resolution is inferred from the size of the image. Manual calibration can be done by gathering the three types of information by hand and passing them directly to the manual calibration function *scroll_calibration_manual*. It is recommended to use automatic calibration, as it is easier and more reliable compared to manual calibration. The latter should be reserved for when *eyeScrollR* is used in contexts other than web pages.

**Fig. 2:**
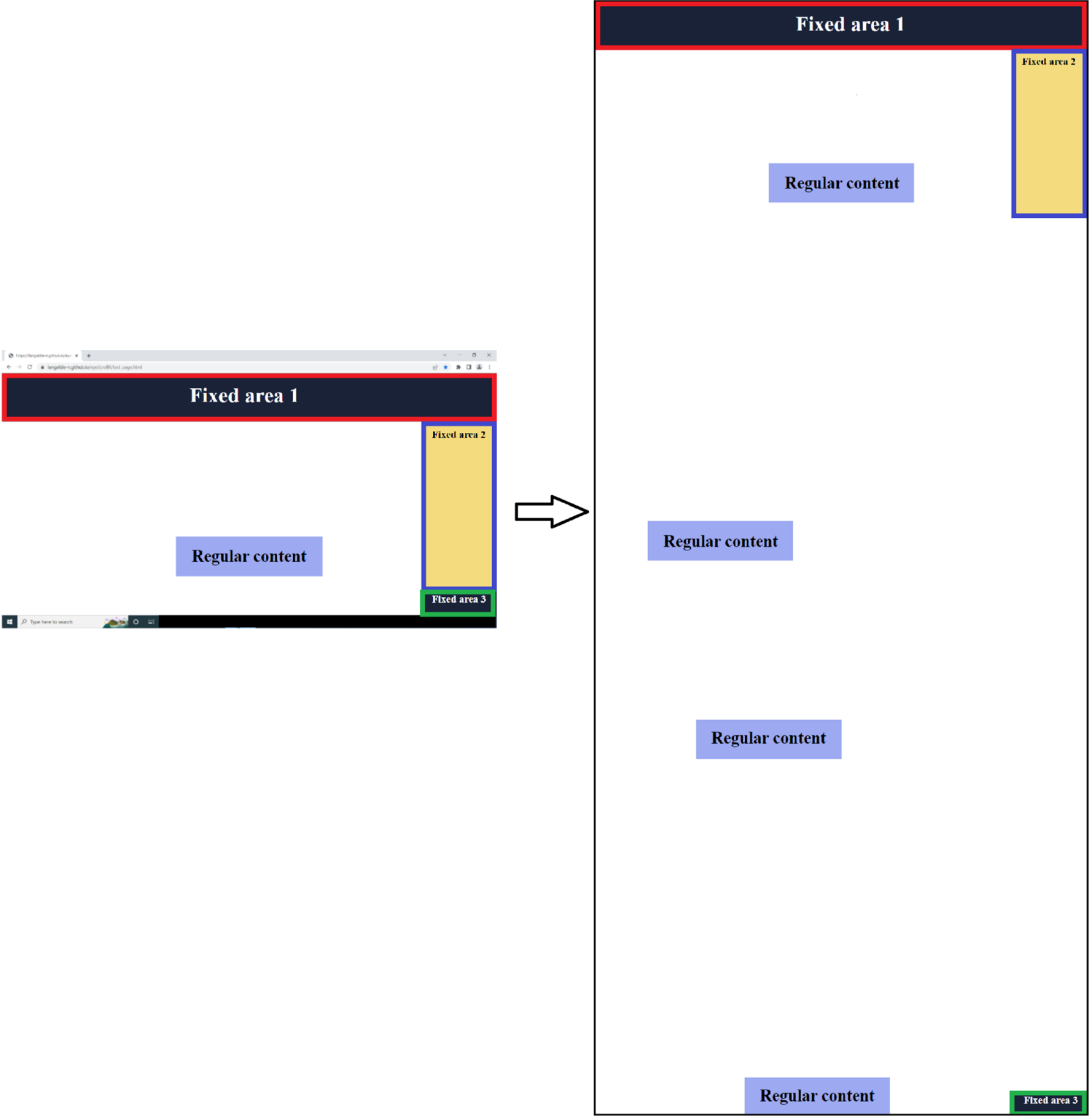
Three rectangular fixed areas on the screen (*left*) are specified, and redirected to the full-page image (*right*)

### Step 3. Eye-tracking data collection

Any combination of eye tracker and software will work with the *eyeScrollR* package, as long as it produces a data set that contains the variables described in Step 6. The only critical point is that the participants use the browser prepared for the study in Step 1. The eye-tracking data collection can also take place after Step 4.

### Step 4. Get full-page image

This step consists of getting the full-page image of the web page to which gaze data will be mapped. Its purpose is to: a) give the user a visual representation of the complete web page, b) get the total length of the web page, c) produce measurements of fixed areas if there are any (see Step 5), and d) optionally create a heatmap (see Step 7). There are several plugins for standard browsers and pieces of software that can produce full-page images of scrollable web pages (e.g., “GoFullPage” for Chromium browsers). Any tool is acceptable, as long as the resulting full-page image has the same pixel width as the viewing area and contains all visible content. Note that complex and dynamic web pages may require the researcher to perform some manual editing of the full-page image to ensure fidelity with the web page as it appears during browsing.

### Step 5. Specify locations of fixed areas on the full-page image (optional)

Some web pages contain fixed areas, which means that the content does not scroll up or down with the rest of the content. A typical example of fixed areas is a menu at the top of a web page, a sidebar, or advertising that stays in place so that it is always visible to the user. A key feature of the *eyeScrollR* package is the possibility to specify such fixed areas. Specifying these areas will inform the package not to apply any scroll-based correction to gazes and fixations that are recorded inside the fixed areas. Instead, gazes and fixation inside fixed areas are redirected to the specified locations on the full-page image acquired in Step 4. The specification of fixed areas is done by manually mapping rectangular areas on the screen to specific areas on the full-page image, as shown in Fig. 2.

Sometimes the appearance of fixed areas changes during user interaction. For instance, top menus sometimes retract or disappear after a certain number of pixels have been scrolled, when a button has been clicked, and so on. To accommodate such situations, the *eyeScrollR* package handles fixed areas with *bundles* and *rules*. In *eyeScrollR* terminology, a bundle is a set of one or more fixed areas corresponding to a given web page configuration. A *rule* is attached to each bundle to specify conditions when the bundle of fixed areas should or should not be used. As an example, consider a web page with a top menu (Fixed area 1) and a sidebar (Fixed area 2), similar to the one in Fig. 2, which do not move or change when the user is scrolling down. The top menu and the sidebar are two fixed areas that together comprise a bundle, each with a manually specified location on the full-page image, and an associated rule specifying that this bundle (and therefore all of its fixed areas) is always active.

Another possible situation is that the top menu and the sidebar disappear when the user has scrolled down more than 1000 pixels, but reappear if the user scrolls up again. In this situation, the bundle should be associated with a rule stating that the bundle is only active when the user has scrolled fewer than 1000 pixels, and inactive otherwise.

As a final example, consider a situation where, instead of completely disappearing, the fixed areas shrink when the user has scrolled down more than 1000 pixels. This implies that the web page has two different configurations, both with their own slightly different coordinates of fixed areas. Each one of these configurations leads to a different bundle: one bundle that specifies the first configuration (with its own fixed areas and their specified locations on the full-page image) and is associated with a rule making it active when the user has scrolled fewer than 1000 pixels and inactive otherwise, and another bundle that specifies the second configuration and is associated with a rule making it inactive when the user has scrolled fewer than 1000 pixels and active otherwise.

In practice, rules are custom R functions that return the logical value TRUE when the coupled bundle must be active, and the value FALSE when the bundle must be inactive. The necessary code for the creation of bundles and rules can be demanding for researchers with limited programming experience. To reduce the need for programming experience, the *eyeScrollR* package contains a Shiny gadget interface that outputs the necessary code. However, only a handful of the most common rules have been pre-specified in the gadget, and it may be necessary for some users to write their own code. More details about how the bundles of fixed areas and their related rules work can be found in Appendix A.

### Step 6. Load data set and correct

This is the critical step in the method, in which all of the previous steps’ data are used to map screen coordinates from the eye tracker to full-page image coordinates. The mapping is performed by calling the *eye_scroll_correct* function. It requires a correctly formed and chronologically ordered data set, including at least the following columns with these exact names:

- *Timestamp*: Timestamp for each row of data.
- *Data*: Column including events, such as mouse scroll, key presses, or mouse clicks. The column must contain a string value when the user scrolls which consist of key:value pairs separated by semicolons as follows: X:{mouse *X*-coordinate}; Y:{mouse *Y*-coordinate}; MouseEvent:WM_MOUSEWHEEL; ScrollDelta:{positive or negative integer value indicating respectively a scroll up or down}. The mouse *X*- and *Y*-coordinates are necessary since usually, scroll events do not affect web page scrolling when the mouse cursor is placed outside the viewing area, for instance, if the user tries to scroll while the mouse is placed over the browser tabs. Generating a data column that contains scrolling input will be different for each data collection method. Some eye-tracking software will directly output this column using the right structure. In other cases, it may be necessary to concatenate or rewrite the relevant pieces of information from different columns or sources to fit this structure of key:value pairs separated by semi-colons, and/or join this column with the rest of the eye-tracking data by timestamps.^1^
- *Gaze.X* and *Gaze.Y*: *X*- and *Y*-coordinates of gaze data (mandatory if there are no fixation data).
- *Fixation.X* and *Fixation.Y*: *X*- and *Y*-coordinates of fixation data (mandatory if there are no gaze data). Each row must have timestamps that match the gaze points composing the fixations. Some eye-tracking software can directly output data in this form, while others will require the user to join data sets by timestamps or fixation ID.^2^

A short extract from a correctly formed data set including a mouse scroll event is shown in Table 1. The *eye_scroll_correct* function returns a data set that includes all original columns plus the following new ones:

- *Scroll*: How many pixels have been scrolled up or down at this timestamp.
- *Timestamp.Shifted*: The timestamps, shifted by the value of the *time_shift* argument.
- *Corrected.Gaze.X* and *Corrected.Gaze.Y*: The gaze coordinates mapped to the full-page image (if gaze data were included in the data set).
- *Corrected.Fixation.X* and *Corrected.Fixation.Y*: The fixation coordinates mapped to the full-page image (if fixation data was included in the data set).

**Table 1:** Extract from a correctly formed data set including both gazes and fixations

Timestamp	Data	Gaze.X	Gaze.Y	Fixation.X	Fixation.Y
$\vdots$	$\vdots$	$\vdots$	$\vdots$	$\vdots$	$\vdots$
55080	*NA*	803	384	783	380
55089	*NA*	794	375	783	380
55095	X:1478;Y:399; MouseEvent:WM_MOUSEWHEEL; ScrollDelta:-120;	*NA*	*NA*	*NA*	*NA*
55097	*NA*	805	387	783	380
$\vdots$	$\vdots$	$\vdots$	$\vdots$	$\vdots$	$\vdots$

When called, the *eye_scroll_correct* function starts by shifting the entire *Timestamp* column of the data set by the optional *time_shift* argument passed to the function. This is to ensure that the researcher can synchronize a data set to a desired timestamp. If the optional *starting_scroll* argument is passed to the function, it will also consider that this number of pixels had already been scrolled down before it starts iterating over the data set.

Every line of data before the *timestamp_start* and after the *timestamp_stop* arguments is removed from the gaze-mapped data set. All remaining *X*- and *Y*-coordinates are translated to match a coordinate system with an origin at the top left corner of the viewing area. If the *outside_image_is_na* argument is set to TRUE, every fixation or gaze that falls outside the viewing area will be set to NA in the resulting gaze-mapped data set.

The function then iterates over the data set line by line. During each iteration, the function first checks if a mouse wheel event has been recorded, and, if so, adds or subtracts the *scroll_pixels* value (obtained in the calibration step) to the total number of pixels scrolled from the top of the web page.

If the *scroll_lag* argument is passed to the function, changes in the y-coordinate are delayed (lagged) by the specified amount. The argument is optional but recommended to increase accuracy, as changes on the monitor never happen immediately when an input message is received. Since monitors have a finite refresh rate and computational resources are limited, a change cannot be visible immediately. The average input lag is hardware and software-dependent and can be difficult to measure accurately, but it can be approximated. If the temporal distance between an input and the next video frame is assumed to be uniformly distributed between 0*ms* and the monitor frame duration, then on average, the next frame will be displayed half of that time after any given input. Any frame after the very next one will then be shifted by entire frames duration. Hence the equation:

$$\begin{aligned} scroll\_lag = (n\_frame-1)\cdot \frac{1000}{refresh\_rate} + \frac{1}{2}\cdot \frac{1000}{refresh\_rate} \end{aligned}$$

With $n\_frame$ being a positive integer indicating the rank of the next monitor frame on which the change is assumed to happen on average, and $refresh\_rate$ the monitor refresh rate. As an example, assuming that inputs tend to be visible on the next 60-Hz monitor frame ($n\_frame=1$), *scroll_lag* should be set to 8.333*ms*, and this is the minimum recommended for 60 Hz monitors. Any number below 50 ms should in general be reasonable ($n\_frame=\{1, 2, 3\}$ for a 60 Hz monitor), but as a general rule, $n\_frame=2$ is recommended. The user can use the *get_scroll_lag* function to get a recommended argument as a function of $refresh\_rate$ and $n\_frame$.

The *eye_scroll_correct* function then checks individually for each rule in the *rules* argument if, within the current conditions, they are true or false. The function then checks for each rule that returns TRUE if the current data point is within one of the fixed areas in its associated bundle. If so, the data point is translated to its corresponding area in the full-page image. If it is not, then the data point Y coordinate is corrected by adding the current number of pixels scrolled from the top.

Once all lines in the data set have been read and if the *output_file* argument is not an empty string, the function writes the resulting data set in this file name in csv format.

### Step 7. Output heatmap (optional)

*eyeScrollR* offers a heatmap generating function that produces a heatmap from the full-page image created in Step 4, and a single data set that has been mapped by the core function in Step 6. Inspecting the heatmap is useful for ensuring that the method has produced a valid mapping of the eye-tracking data to the full-page image.

## A reproducible example

In this section, we describe a reproducible example that demonstrates how to use the *eyeScrollR* package in each of the seven steps of the workflow for a single participant, including commented code snippets. The web page used in this example can be found at https://larigaldie-n.github.io/eyeScrollR/test_page.html. Please keep in mind that simply copying and pasting our code may not produce the expected result, as several values may need to be adapted to a specific work environment. The example is complex enough to demonstrate all functionalities of the package, as the web page contains several fixed areas, some of which change in size when the user has scrolled down a certain amount. Please note that there is another example and a different version of this reproducible example, as well as complete documentation, accessible at https://larigaldie-n.github.io/eyeScrollR/.

### Step 0. Install and load the package

To install and load *eyeScrollR*, first install and source the *devtools* package, then install the *eyeScrollR* package from the GitHub repository, and finally load the package:
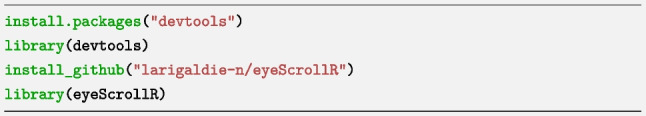


### Step 1. Prepare the study setting

In this example, we used the Chrome browser, so we deactivated the ’Smooth Scrolling’ and enabled the ’Overlay Scrollbars” options in the ’chrome://flags/’ menu in order to disable smooth scrolling and hide side scrollbars. We used a Logitech M310 mouse, with a standard mouse wheel that scrolls the same amount every time when turned. Finally, since we designed the web page, we knew that getting the full-page image would only require the use of a browser plugin at our convenience.

### Step 2. Calibrate scrolling

We used the automatic calibration. We visited the calibration page https://larigaldie-n.github.io/eyeScrollR/calibration.html on the same computer and browser as the participant in Step 3. We took a screenshot using the “Snip & Sketch” tool in Windows. We saved the screenshot as “calibration_image.png” in the R working directory. We manually read the number of *scroll_pixels* from the screenshot - the value was 125. We then typed the following code in R:
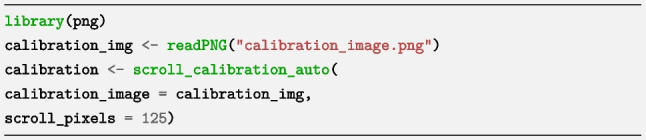


### Step 3. Collection of eye-tracking data

In this example, one participant browsed the web page https://larigaldie-n.github.io/eyeScrollR/test_page.html. The iMotions software was used to record and export eye-tracking data. iMotions is an experiment builder and data collection software that allows one to run studies, log inputs, record the screen, and perform data analysis and visualization. It can integrate several biosensors, including eye trackers. A Smart Eye Aurora eye tracker with a 120-Hz refresh rate was used in this example.

### Step 4. Get full-page image

We obtained the full page by using the free extension “Make screenshot for Chrome” for the Chrome browser. We saved the image as “example_website.png” with the default dimensions 1920x4377 in the R working directory.

### Step 5. Specify locations of fixed areas on the full-page image

An examination of the web page https://larigaldie-n.github.io/eyeScrollR/test_page.html revealed that the top, right, and bottom right areas all remain visible when the user scrolls, indicating that these three areas are fixed areas. However, after a certain number of pixels have been scrolled, the top and right areas change in size. This implies two different web page configurations and the need for two different bundles of fixed areas. In this step, we map the three fixed areas to the full-page image in the two bundles, and specify rules using the bundles gadget to activate and deactivate each bundle when appropriate. We opened the bundles gadget by typing the following code in R:
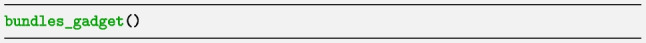


The code opens the gadget in the R Studio Viewer. We click on the button “Add new bundle” and then on the button “Add fixed area in Bundle”. The gadget requires the following information for fixed area 1: *X*-*Y* coordinates of the top left and bottom right of the fixed area 1 on the screen and *X*-*Y* coordinates of the top left and bottom right of the fixed area 1 on the full-page image. The process is then repeated for fixed areas 2 and 3 inside the first bundle: we click “Add fixed area in Bundle”, then input the *X*-*Y* coordinates of the top left and bottom right of areas on the screen and on the full-page image.

The first bundle of fixed areas used the rule “True when Scrolled < value” to indicate that the configuration of fixed areas in the bundle should be applied until a certain number of pixels have been scrolled down from the top.

Determining the number of pixels at which the change in fixed areas occurs requires some investigation - for instance, counting the number of scrolls (the number of notches on the scroll wheel that is turned) until the web page configuration changes, and inferring the corresponding number of pixels with the help of the calibration page. In our case, the Fixed area 1 (top area) shrinks when 1000 pixels or more have been scrolled down and expands when the page has been scrolled up again until a total of less than 1000 pixels have been scrolled. The gadget automatically generated the following code for the first bundle (we only changed the value in the rule to suit our needs):
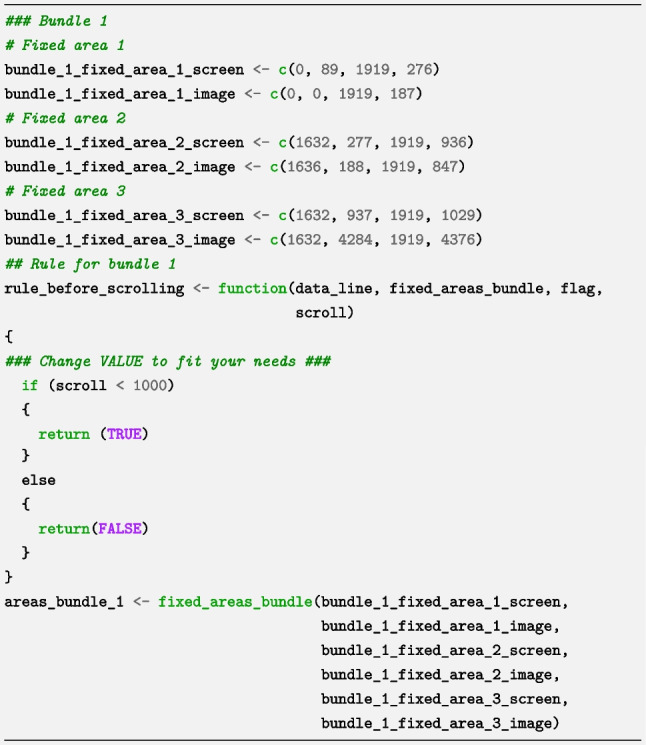

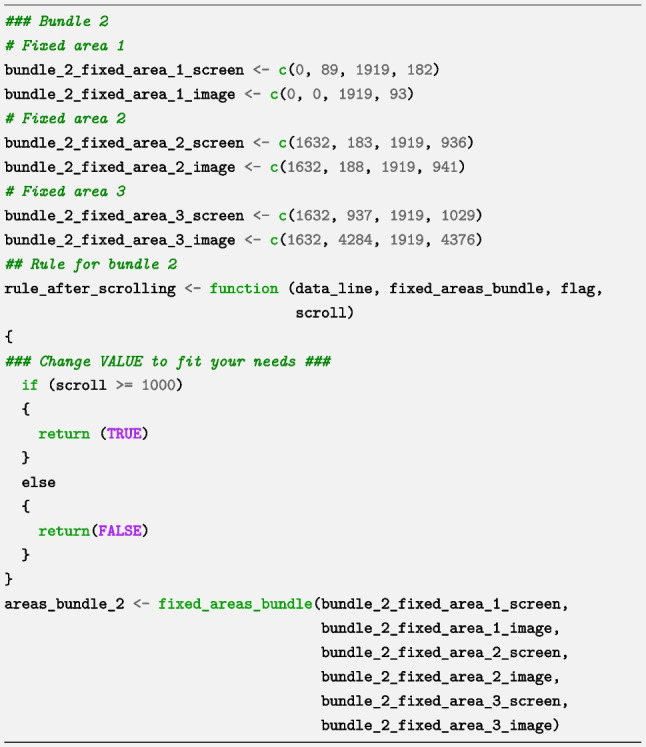


We then built a new bundle by choosing “Add new bundle”, and in this second bundle, we specified the fixed areas in the second web page configuration. This second bundle (visible after scrolling more than 1000 pixels down) produced a different set of measurements of fixed areas and required a different rule to specify that this bundle should only be used after 1000 pixels had been scrolled (which corresponds to the rule “True when Scrolled >= value”). In other words, by using this rule, we ensured that the specified locations of all fixed areas in the second bundle were not active when less than 1000 pixels were scrolled, but were active when more than 1000 pixels were scrolled. For this second bundle, the gadget generated the following code (we only changed the value in the rule to suit our needs):

Since the core function requires all bundles and rules to be put in lists, the generated code ends as follows:
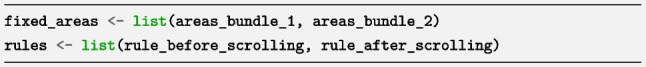


Although the code in this section appears to be lengthy, it is almost always auto-generated by the *bundles_gadget* function and only needs to be done once per web page containing fixed areas.

### Step 6. Load data set and correct

The next step is to import the data set with correct column names and content^3^. We extracted the data from the iMotions software in the following way. We clicked on the button “Add analysis” and selected the participant and the recording of web page browsing. Then, we clicked on the buttons “Export” - “Sensor data” - “Export”. In the tab “Respondents” we selected our participant. In the tab “Stimuli” we selected the browsing gaze recording. In the tab “Sensors” among “R Analysis GazeAnalysis I-VT filter” options we selected “X-coordinate of fixation” and “Y-coordinate of fixation”. In the tab “File info” we selected “user-generated events” Then, we clicked on the button “Export” in the bottom right. We save the dataset as “data.csv” in the R working directory. We load the data with the following code:
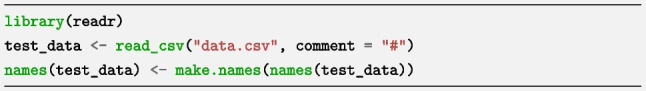


We want *eyeScrollR* to map fixations within the period when the web page was fully loaded and we therefore need to obtain the timestamp when the web page was fully loaded and the timestamp when the participant finished browsing it. We obtained the starting and ending timestamps when the user was browsing manually as 3577 and 30864 by watching the gaze screen recording. We also manually obtained the width and height of the full-page image (1920*4377 pixels) by right-clicking on the image and then on “properties”. We then typed the values mentioned above in the *eye_scroll_correct* function, and to account for input lag on a 60-Hz monitor, we set the *scroll_lag* argument using the *get_scroll_lag argument with n_frames = 2*:
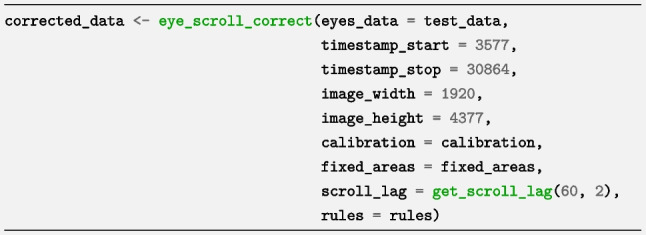


The objects “calibration”, “fixed_areas”, and “rules” were created in Steps 2 and 5.

### Step 7. Output heatmap

We loaded the full-page image “example_website.png”, and produced a heatmap with the *generate_heatmap* function:
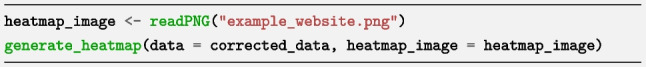


Appendix B contains the resulting heatmap from our example.

## Validation study

To assess the validity of the *eyeScrollR* package, four different web pages were browsed by a participant in four different hardware and software settings. A random sample of *eyeScrollR* fixation points was chosen and compared to manual coding of the same data. The participant navigated the web pages, insisting on potentially limiting cases (e.g., fast scrolling patterns, scrolling more than the total length of the image, insistent gaze at all fixed areas when relevant). The participant had no time limit and simply navigated web pages long enough to collect data from both basic and complex mapping situations. Different hardware and software settings were used in order to test the robustness of *eyeScrollR* and its usefulness in combination with commercial software (iMotions) as well as open-source software (Python). A Chrome browser was used in all study settings. The four study settings were the following:

- We used a desktop computer using the SR Research EyeLink 1000 eye tracker with a sample rate of 1000 Hz, and a 19” monitor with native resolution 1680 x 1050 and a 60-Hz refresh rate. The study was run using custom Python code for input and 30-fps screen recording, and to launch Google Chrome with the correct web page. The eye tracker itself was piloted using the SR Research PyLink Python library version 2.1.762.0.
- We used the same hardware and software settings as above but replaced the monitor with a 24” one with native resolution 1920 x 1080 and a 60-Hz refresh rate.
- We used the same hardware and software settings as above but replaced the monitor with another 24” one with native resolution 1920 x 1200 and a 60-Hz refresh rate.
- We used a laptop computer with the Aurora Smart Eye eye tracker with a sample rate of 120 Hz, with a 15.6” monitor with a 1920 x 1080 resolution and a 60-Hz refresh rate. The study was run using iMotions version 9.3 for input, 24-fps screen recording, and piloting the eye tracker. Google Chrome was opened manually on the correct web pages.

The four web pages were the following:

- “test page 1”: A custom web page designed for new users to test *eyeScrollR* in the absence of fixed areas (https://larigaldie-n.github.io/eyeScrollR/test_no_fixed.html)
- “test page 2”: A custom web page designed for new users to test *eyeScrollR* in the presence of fixed areas (https://larigaldie-n.github.io/eyeScrollR/test_page.html)
- “PsychoPy”: The PsychoPy homepage (https://psychopy.org)
- “OSF”: The OSF homepage when not logged in (https://osf.io)

This resulted in 16 different situations (4 settings x 4 web pages). After data collection, the raw eye-tracking data containing all gaze and fixation coordinates, timestamps, and user input were exported. The EyeLink Data Viewer does not automatically output a data file that combines gaze and fixation and an R script was written to merge gaze and fixation data and synchronize it with video and input recordings. All data points were then gaze-mapped separately for each full-page image using *eyeScrollR*. All full-page images were created with the free extension “Full Page Screenshot” for the Chrome browser and were slightly modified if necessary to include all areas from the web pages.

Within each situation, *eyeScrollR* was run with a conservative 8.333*ms* *scroll_lag* argument. Thirty *eyeScrollR*-mapped fixations lasting longer than two video frames were randomly selected for manual coding. The reason for using fixations lasting longer than two video frames was to limit the chances of the fixations not being captured by the video recording (for details see the Discussion below). One fixation was removed due to an incorrect initialization of the web page presentation during data collection, resulting in a total of 479 randomly selected data points. The manual coding was performed by two coders who were blind to the *eyeScrollR* mapping. To perform the manual coding on the full-page image, the coders were given the set of fixation coordinates on the screen, the full-page image, and for each fixation a frame extracted from the video at the correct timestamp with a red pixel indicating the location of the fixation. The video frame was extracted at the median timestamp for each fixation. All data, code, and materials for the validation study are available at https://osf.io/6e8sr/.

**Fig. 3:**
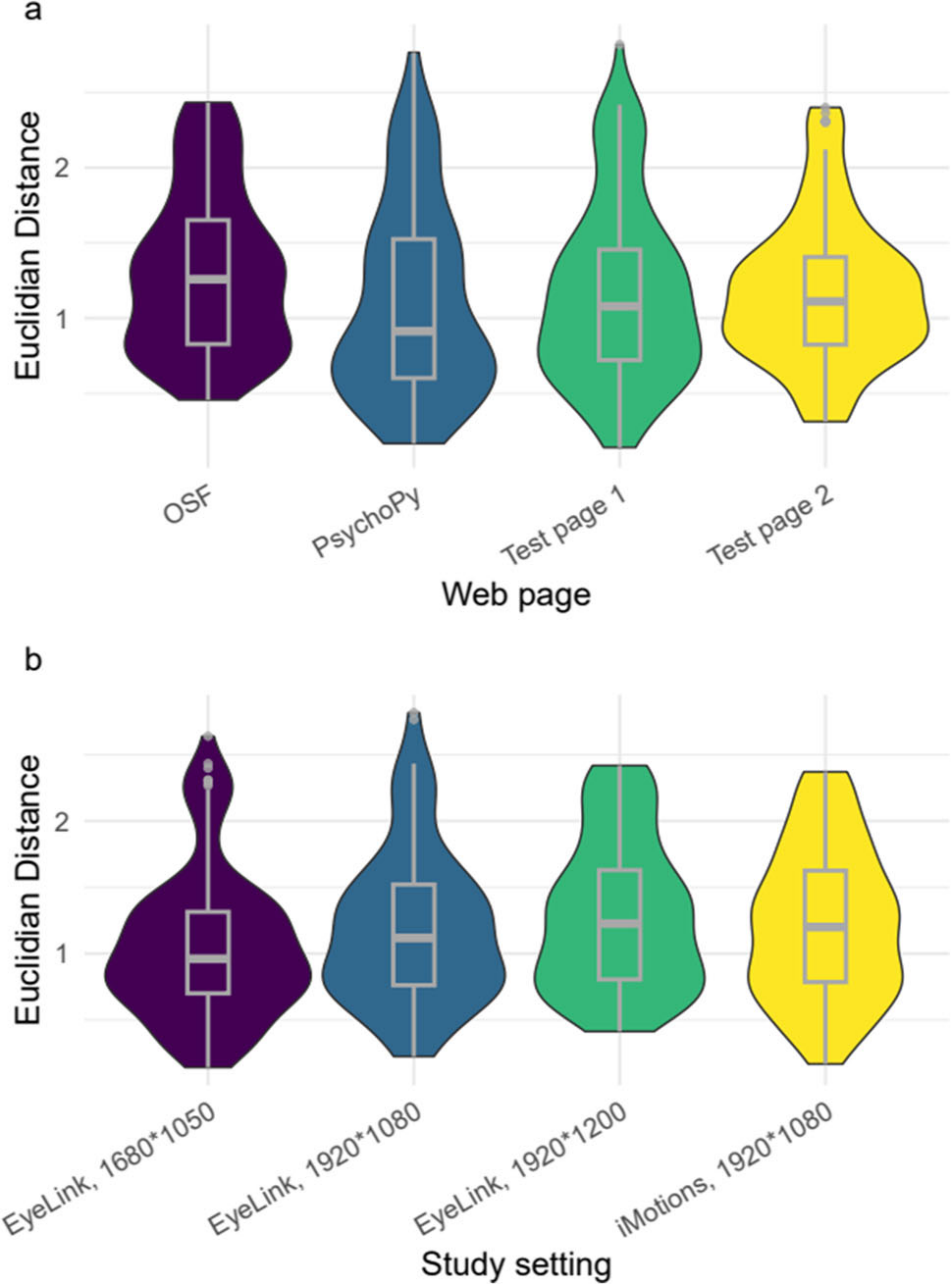
Violin and box plot of Euclidean distances between *eyeScrollR* mappings and manual coding by web page (**a**) and study setting (**b**). Distances larger than 3 pixels (eight data points out of 479) are excluded for readability

### Results

We calculated the Euclidean distance between all pairs of *X*-*Y* coordinates mapped by manual coding and *eyeScrollR*. All Euclidean distances greater than or equal to 3 pixels were further examined to determine if the discrepancy was due to *eyeScrollR* inaccuracies or manual coding. The examination revealed that the manual coders had made 47 errors out of 479 data points (9.81%, mean difference of 689.97 pixels). It should be noted that even without coding errors some irreducible inaccuracy is expected. This is due to the fact that eye trackers (and thus *eyeScrollR*) return continuous coordinates, which cannot be correctly mapped by a manual coder working with discrete pixel coordinates. After correcting all manual coding errors, only eight differences larger than 3 pixels remained (1.67%, mean difference of 112.37 pixels) that could be due to *eyeScrollR*. The remaining errors were all consistent with a shift of exactly one (seven data points with a distance in the range [97; 103] pixels) or two (one data point with a difference in the range [197; 203] pixels) mouse scrolls. The distribution of Euclidean distances between *eyeScrollR* and manual coding for all study settings and web pages are shown in Fig. 3. The descriptive statistics are shown in Table 2.

**Table 2:** Descriptive statistics of Euclidean distance between *eyeScrollR* mappings and manual coding, excluding distances larger than 3 pixels (eight data points out of 479)

Condition		Mean	SD	Median
Web page	OSF	1.31	0.53	1.26
	PsychoPy	1.11	0.64	0.91
	Test page 1	1.17	0.59	1.08
	Test page 2	1.18	0.48	1.11
Setting	EyeLink, 1680*1050	1.06	0.55	0.96
	EyeLink, 1920*1080	1.19	0.57	1.12
	EyeLink, 1920*1200	1.29	0.56	1.23
	iMotions, 1920*1080	1.23	0.56	1.2
Total		1.19	0.57	1.1

### Discussion

The results show that *eyeScrollR* produces gaze-mappings that are on average very close to manual coding and differences between the two methods are generally no greater than 1–2 pixels. The results are very similar across different web pages, hardware, and software settings. The manual coding resulted in human coding errors in 9.81% of the data, with an average distance of 689.97 pixels while potential software errors were present in 1.67% of the data, with an average distance of 112.37 pixels. Among the eight differences larger than 3 pixels, one was unambiguous due to a video recording inaccuracy. In the study setting using the Aurora eye tracker with the iMotions data collection software, the recorded video frame number 2658, starting at timestamp $t=111022 ms$, shows a change on the screen corresponding to a shift of a unique down scroll. However, a manual examination of the data file reveals that the timestamp at which the scroll occurred was $t=111163 ms$ that is, 141*ms* after the visible change in the video, during video frame number 2661. In other words, the video recording shows a change that could not have occurred on the participant’s monitor for at least another 141*ms*. The random fixation point selected for manual coding occurred during frame number 2660, beginning at $t=111113 ms$, 50*ms* before the mouse scroll event. Consequently, the manual coding was performed on a frame that was ahead of the monitor display. On the other hand, *eyeScrollR* did not consider that any change occurred until a few milliseconds after the scrolling event (with an offset corresponding to the *scroll_lag* argument of 8.33*ms* passed to the core function).

This situation points to a general challenge with manual coding and other gaze-mapping methods based on video recording. Video recordings typically have fewer frames per second than what is actually displayed on a monitor. Therefore, they are not a real-time representation of what happened during the study and should not be treated as such. Consider a video frame captured at $t=0 ms$ in a 24-fps video, with a fixation and a scroll starting at the same timestamp. Due to the scroll, the display will change during the next 60-Hz monitor refresh, at $t=1 ms$, but the video will only change on its next frame, at $t=41.67 ms$. A manual coding will map the fixation coordinates for the duration of the video frame, and will therefore be incorrect for 40.67*ms*. If *eyeScrollR* performed its mapping assuming that the display changed at $t=0 ms$, the manual coder would conclude that *eyeScrollR* made an error of one scroll worth of pixels. While *eyeScrollR* would be incorrect for 1*ms*, it would still be correct for the rest of the time; something that the manual coder cannot check. A slight change in the scenario produces even worse results: if a fixation starts at $t=1 ms$ instead of at $t=0 ms$, a manual coder would still work on the video frame starting at $t=0 ms$. Furthermore, this reasoning assumes maximally accurate video recordings with no frame skipping or belating caused by fluctuations in computational power, for example. In the presence of computer lag, inaccuracies from video-based gaze-mapping methods could easily reach 100*ms* or more. These unavoidable issues mean that very short fixations can occur on frames that were displayed on the monitor but never recorded on video. In order to reduce such occurrences in our study, we excluded all fixations shorter than two video frames from the analysis above.

On the other hand, by using *eyeScrollR*’s *scroll_lag* argument, a user can delay the scroll corrections based on a fixed timing. Assuming that scrolls tend to be displayed on the following monitor frame, setting a *scroll_lag* to half of the monitor frame duration ensures that *eyeScrollR* will, on average, start its scroll correction at the right timestamp. In this study, all distances larger than 3 pixels attributed to *eyeScrollR* were in the near vicinity of mouse scroll input messages and a video frame change, similar to the ambiguous situation described here. This suggests that we cannot know for sure whether it was *eyeScrollR* or manual coding that was incorrect. However, we believe that because *eyeScrollR* does not suffer low frame resolution, potential frame lag, or frame skipping it will on average make fewer and shorter errors than any video-based method, whether manual, assisted, or automatic.

## General discussion

This article introduces *eyeScrollR*, an open-source R package for gaze-mapping eye-tracking data with screen coordinates to full-page image coordinates. The validation study showed that, despite a few discrepancies, *eyeScrollR* and manual coding have a very high agreement.

After a manual inspection, we discovered that the vast majority of consequential differences between the methods were due to human errors. As irreducible temporal inaccuracies are expected in both methods, none of the remaining errors could be unambiguously attributed to *eyeScrollR*. All significant differences between the methods were a multiple of a mouse scroll and could either be due to *eyeScrollR* or to video frames being behind or slightly ahead or lagging behind the true display. All in all, temporal accuracy should be expected to be slightly better for *eyeScrollR* than manual coding. However, users should be aware that the temporal accuracy can never be perfect using either *eyeScrollR* or video-based mapping methods, especially when scrolling happens in the middle of a fixation.

Of all currently known methods for gaze-mapping in scrolling situations, *eyeScrollR* is the only one that is both reproducible and transparent. Furthermore, as free software, it is available to all researchers, including those who cannot afford expensive alternatives. Code availability and open license facilitates collaborative evaluation and improvement and allow researchers to predict its possible uses and limitations. This makes it an ideal tool for researchers who wish to adhere to open science principles. Finally, for researchers who are familiar with R, *eyeScrollR* is easy and fast to use thanks to its Shiny gadget (Chang et al., 2022) and online tutorials.

**Fig. 4:**
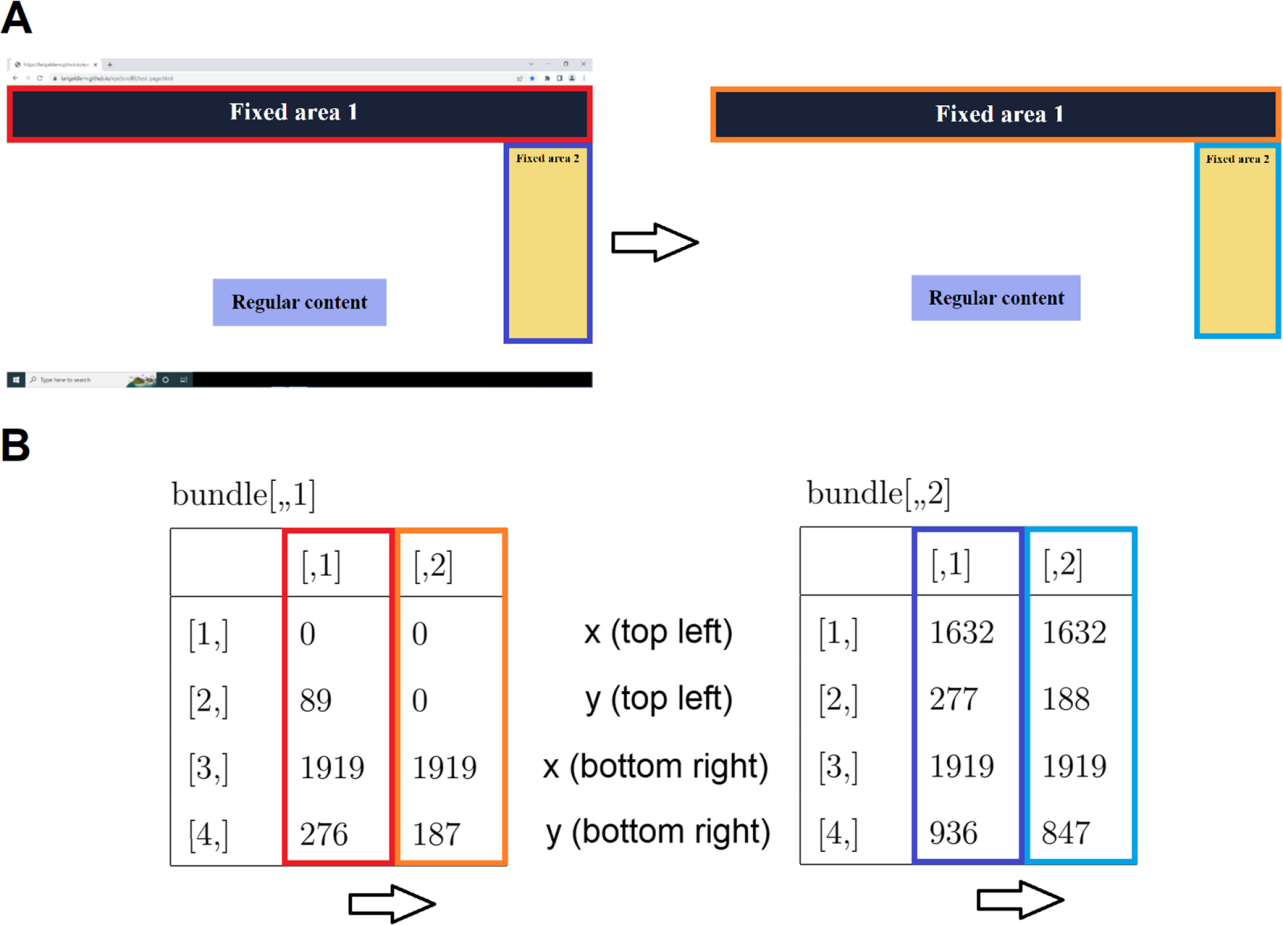
**A**) Two rectangular fixed areas on the screen (*left*) are specified, and redirected to the full-page image (*right*). **B**) 3D array representing the bundle containing these two fixed areas. Rows are *x* and *y* values from the top left and bottom right pixels. The first column represents the coordinates of the fixed area on the screen, and the second column the coordinates of the fixed area on the full-page image. Each layer (left and right) represents a fixed area specification, with the coordinates of the redirection from the screen to the full-page image

Although *eyeScrollR* allows researchers to apply coordinate corrections to both gaze and fixation data, it is recommended that fixations and saccades are inferred from raw gaze data before using the package, particularly for web pages that include fixed areas. If fixations and saccades are inferred after using *eyeScrollR*, gaze clusters positioned between a fixed and a scrollable area could result in gazes being corrected only for those in the scrollable area, and may be interpreted as either unrealistic saccades or as two different fixations. Similarly, stationary gaze clusters during a scroll will be interpreted as saccades if the correction is applied before inferring fixations. It should also be noted that web pages that include subcomponents with their own scrollbars (e.g., Overleaf or Gmail) may not work properly due to the fact that *eyeScrollR* will interpret all mouse scrolls in the browsing area as scrolling on a unique full-page document. Other methods of scrolling up and down (e.g., the “page up” and “page down” keys) are not yet supported, which may necessitate researchers to either deactivate those keys or simply instruct participants not to use them. The same logic applies to using a scrollbar directly to move the content.

The complete code for the *eyeScrollR* package is available at https://github.com/larigaldie-n/eyeScrollR, the code, data, and materials for the validation study are available at https://osf.io/6e8sr/. The validation study does not use confirmatory inferential statistics, so it was not preregistered.

## Appendix A: Technical details on fixed areas and rules

The *fixed_areas* list optionally passed to the *eye_scroll_correct* function is a list of bundles of fixed areas on the screen to be mapped to a specific area on the full-page image. Each bundle is a three-dimensional array within which each layer defines a single fixed area on the screen and its respective mapped area on the full-page image. Therefore, each layer is a 2$\times$4 matrix comprised of the *X*- and *Y*-coordinates of the top left and bottom right pixels of the rectangle on the screen where the area is located, and the *X*- and *Y*-coordinates of the top left and bottom right pixels of the same rectangle on the full-page image (see Fig. 4B for a visual example). All bundles are inserted into a list before being passed to the core function.

To control when a bundle is used, a list of *rules* can be passed to the function. This is optional, and if left empty, the program will apply each bundle to the complete data set. Each rule is a function that must return a logical value: TRUE if the associated bundle of fixed areas should be applied and FALSE otherwise. Rules must be written by the researcher, but are called automatically by the core function before each line of the data set is processed. The most common rules are included in the bundle gadget and only require some minor modifications before being used. The following arguments will be passed to every rule function and can therefore be used when writing the function to ascertain if a condition for the bundle to be TRUE or FALSE has been met:

- *data_line*: the line of data that is being iterated through
- *fixed_areas_bundle*: the bundle of fixed areas associated with this rule
- *flag*: a flag stating if the rule is currently TRUE or FALSE
- *scroll*: the number of pixels scrolled from the top of the content

Rules and bundles are linked by their order: the first rule is associated with the first fixed areas bundle, the second rule with the second bundle, etc.

## Appendix C: Alternative data collection, processing, and loading procedure

*eyeScrollR* uses long data files consisting of one row for each input and each sampled gaze point ordered by timestamps. In the reproducible example in this article, the eye-tracking program (iMotions) outputs all columns in the correct format by default. The way data are outputted, however, is determined by the data collection technique and the eye-tracking program used. For example, SR Research’s Data Viewer can generate a sample report with all individually timestamped gaze coordinates in a file, but it cannot automatically associate those rows with the fixation coordinates and instead generates a separate data file with a single row for each fixation. As a result, it is sometimes necessary to do some data processing to create a suitable data set for *eyeScrollR*. This Appendix section describes the key points from the data collection, processing, and loading procedures in the validation study using the EyeLink 1000. It can be used as a starting point and adapted for different settings. The complete code can be found at the OSF repository: https://osf.io/6e8sr/.

### Data collection

In addition to video recording and controlling the eye tracker, the validation study also logs mouse input with custom Python code. This allows for a properly formatted data column and hence the use of *eyeScrollR*. The mouse input logging uses the Pynput library and is only a couple of lines long:

The *start_time* argument sets the timestamp at which the Python script starts. The *queue_csv.put()* command places the timestamp and data columns in a waiting list of rows to gradually write in the outputted csv file. This function can then be used to record events in the main Python code^4^.

### Data processing and loading

Using the Data Viewer, gaze data with the required columns is extracted by clicking “Analysis -> Reports -> Sample Report”, then selecting the variables “TIMESTAMP”, “RIGHT_GAZE_X”, “RIGHT_GAZE_Y” and “RIGHT_FIX_INDEX” (assuming eye tracking was done on the right eye).

Fixation data with the required columns is then extracted by clicking “Analysis -> Reports -> Fixation Report”, then selecting variables “CURRENT_FIX_INDEX”, “CURRENT_FIX_X” and “CURRENT_FIX_Y”.

These data sets are then loaded and merged through the common fixation index in an R script:

The data set now consists of one row per sampled gaze point, with its gaze coordinates and its associated fixation coordinates when relevant. The only mandatory column missing is the data column. Assuming that the data column is outputted in its own csv file and that its timestamps are synchronized with the eye-tracking data, the final step is to combine the two files:

Synchronizing timestamps between input and eye-tracking data was achieved by sending a SYNC_TIME message to the eye tracker during the study, and extracting the information from a Message report in the Data Viewer. Once again, this is demonstrated in the study and data analysis at the OSF repository: https://osf.io/6e8sr/

## Footnotes

1 As an example, the reader can find a Python mouse input logger that automatically creates a correctly formed data column and an R script performing the merge between the input data and an eye-tracking data set from EyeLink in Appendix C, and as part of the code used for the validation study on the OSF repository: https://osf.io/6e8sr/

2 An example of how to do this for EyeLink can be found in Appendix C, and in an R script as part of the validation study on the OSF repository: https://osf.io/6e8sr/

3 The procedure for getting correctly formed data depends on the hardware and software used and will often differ from this example. To help the reader, Appendix C describes the key points for an alternative procedure for data collection, processing, and loading used in the validation study. Furthermore, all Python code for the validation study with EyeLink 1000 and R code for data processing and analysis can be found on the OSF repository: https://osf.io/6e8sr/
